# Association between the DOCK7, PCSK9 and GALNT2 Gene Polymorphisms and Serum Lipid levels

**DOI:** 10.1038/srep19079

**Published:** 2016-01-08

**Authors:** Tao Guo, Rui-Xing Yin, Feng Huang, Li-Mei Yao, Wei-Xiong Lin, Shang-Ling Pan

**Affiliations:** 1 Department of Cardiology, Institute of Cardiovascular Diseases, the First Affiliated Hospital, Nanning 530021, Guangxi, China; 2 Department of Molecular Genetics, Medical Scientific Research Center, Nanning 530021, Guangxi, China; 3 Department of Pathophysiology, School of Premedical Sciences, Guangxi Medical University, Nanning 530021, Guangxi, China

## Abstract

This study was to determine the association between several single nucleotide polymorphisms (SNPs) in the dedicator of cytokinesis 7 (*DOCK7*), proprotein convertase subtilisin/kexin type 9 (*PCSK9*) and polypeptide N-acetylgalactosaminyltransferase 2 (*GALNT2*) and serum lipid levels. Genotyping of 9 SNPs was performed in 881 Jing subjects and 988 Han participants. Allele and genotype frequencies of the detected SNPs were different between the two populations. Several SNPs were associated with triglyceride (TG, rs10889332, rs615563, rs7552841, rs1997947, rs2760537, rs4846913 and rs11122316), high-density lipoprotein (HDL) cholesterol (rs1997947), low-density lipoprotein (LDL) cholesterol (rs1168013 and rs7552841), apolipoprotein (Apo) A1 (rs1997947), ApoB (rs10889332 and rs7552841), and ApoA1/ApoB ratio (rs7552841) in Jing minority; and with TG (rs10889332, rs615563, rs7552841, rs11206517, rs1997947, rs4846913 and rs11122316), HDL cholesterol (rs11206517 and rs4846913), LDL cholesterol (rs1168013), ApoA1 (rs11206517 and rs4846913), ApoB (rs7552841), and ApoA1/ApoB ratio (rs4846913) in Han nationality. Strong linkage disequilibria were noted among the SNPs. The commonest haplotype was G-C-G-C-T-G-C-C-G (>10%). The frequencies of C-C-G-C-T-G-T-C-G, G-C-A-C-T-G-C-C-G, G-C-G-C-T-A-C-C-A, G-C-G-C-T-G-C-C-A, G-C-G-C-T-G-T-C-A haplotypes were different between the two populations. Haplotypes could explain much more serum lipid variation than any single SNP alone especially for TG. Differences in lipid profiles between the two populations might partially attribute to these SNPs and their haplotypes.

Cardiovascular disease (CVD) remains as the leading cause of morbidity and mortality worldwide, and its prevalence is expected to increase further, which exerts a significant economic burden^1^,^2^. Most of the current prevention strategies are focused on identifying and managing the established risk factors including hyperlipidemia^3^ that can be effectively addressed for individuals and populations suffering from atherosclerosis. It is generally agreed that dyslipidemia is complex and the result of the interactions^4^,^5^ of multiple genes^6^,^7^,^8^ and multiple environmental factors^9^,^10^. Statins are highly effective for lowering low-density lipoprotein (LDL) cholesterol levels and, consequently, cardiovascular event rates. However, statins do not eliminate cardiovascular risk. High triglyceride (TG) level is a significant risk factor for independent cardiovascular disease and is a marker for atherogenic remnant lipoproteins, such as very low-density lipoprotein (VLDL) cholesterol. Additionally, with elevated TG levels, a combination of LDL cholesterol with VLDL cholesterol in the measure of non-high-density lipoprotein (HDL) cholesterol may be a better predictor of cardiovascular risk than LDL cholesterol alone. Therefore, improved understanding of TG-related loci may optimize patient management strategies, provide potential new targets for future individual therapy, and thereby improve patients’ chances for survival.

Candidate gene and genome-wide association studies (GWASs)^11^,^12^,^13^ have identified a number of sequence variants that explain some of the individual variation in the susceptibility for high TG levels. The dedicator of cytokinesis 7 (*DOCK7*; Gene ID: 85440; MIM: 615730) formerly known as *ZIR2* and *EIEE23*, is located on chromosome 1p31.3 and the protein encoded by this gene is a guanine nucleotide exchange factor (GEF) that plays a role in axon formation and neuronal polarization. The encoded protein displays GEF activity toward RAC1 and RAC3 Rho small GTPases but not toward CDC42. Several transcript variants encoding different isoforms have been found for this gene. The proprotein convertase subtilisin/kexin type 9 (*PCSK9*; Gene ID: 255738; MIM: 607786) gene, also known as *FH3*, *PC9*, *NARC1*, *LDLCQ1*, *NARC-1* and *HCHOLA3*, is located on 1p32.3 and this gene encodes a member of the subtilisin-like proprotein convertase family, which includes proteases that process protein and peptide precursors trafficking through regulated or constitutive branches of the secretory pathway. The encoded protein undergoes an autocatalytic processing event with its prosegment in the ER and is constitutively secreted as an inactive protease into the extracellular matrix and trans-Golgi network. It is expressed in liver, intestine and kidney tissues and escorts specific receptors for lysosomal degradation. It plays a role in cholesterol and fatty acid metabolism. Mutations in this gene have been associated with autosomal dominant familial hypercholesterolemia. Alternative splicing can result in multiple transcript variants. The polypeptide N-acetylgalactosaminyltransferase 2 (*GALNT2*; Gene ID: 2590; MIM: 602274) gene, formerly known as *GalNAc-T2*, is located on chromosome 1q41-q42 and encodes a member of the glycosyltransferase 2 protein family. Members of this family initiate mucin-type O-glycoslation of peptides in the Golgi apparatus. The encoded protein may be involved in O-linked glycosylation of the immunoglobulin A1 hinge region. This gene may influence TG levels, and may be involved type 2 diabetes, as well as several types of cancer. Alternative splicing can also result in multiple transcript variants (http://www.ncbi.nlm.nih.gov/gene/).

Human genetic studies of lipid levels can identify targets for new therapies for cholesterol management and prevention of heart disease especially monoclonal anti-PCSK9 antibodies are already on the market to significantly reduced levels of LDL cholesterol when added to statin therapy administered at the maximum tolerated dose^14^. For comparison with the nonsynonymous single nucleotide polymorphisms (SNPs) in known drug therapies genes, we scored point mutations at synonymous point mutations in housekeeping genes or genes of unknown function on the approximate locations of the chromosome 1. The exact positions of the *PCSK9* SNPs were located in the similar position area of the *DOCK7* and *GLANT2 SNPs* (http://hapmap.ncbi.nlm.nih.gov/). Several genetic variants in the *DOCK7*, *PCSK9* and *GLANT2* have been associated with serum lipid parameters, especially with TG in Western populations^15^, e.g. the SNPs of *DOCK7* rs1167998, rs10889353^16^, *PCSK9* rs11591147^17^ and *GALNT2* rs4846914^13^ were associated with TG levels in European and *PCSK9* rs505151^18^ and *GALNT2* rs2144300 and rs4846914^19^ in the Asian populations. However, the association of the *DOCK7* (rs1168013 and rs10889332), *PCSK9* (rs615563, rs7552841 and rs1126517) and *GALNT2* (rs1997947, rs2760537, rs4846913 and rs11122316) SNPs and serum lipid levels has not been previously reported. Since ancient times China is a multi-ethnic country. Among 56 nationalities in China, the Han nationality is the biggest one. Jing is one of the smallest population of ethnic minorities in southern China with a population of 22,517 (in 2000 the fifth national census statistics of China), China’s only a coastal fishery ethnic minority, and China’s only national ocean at the same time^20^. Jing populations live in Dongxing city, Guangxi Zhuang Autonomous Region. Diet to rice is given priority to, fresh fish and shrimp more, like to use fish sauce to taste. The history of Jing ethnic minority shows Jing nationality is a relatively conservative and isolated minority, and preserves their custom of intra-ethnic marriage^21^. Thus, their genetic background may be less heterogeneous within the population. Little is known about the association of SNPs and lipid phenotypes in the Jing population. Therefore, this research was undertaken to detect the association of the *DOCK7* rs1168013, *DOCK7* rs10889332, *PCSK9* rs615563, *PCSK9* rs7552841, *PCSK9* rs11206517, *GALNT2* rs1997947, *GALNT2* rs2760537, *GALNT2* rs4846913, and *GALNT2* rs11122316 SNPs and lipid profiles in the two ethnic groups.

## Results

### Demographic and clinical characteristics

Table 1 summarized the value of weight, waist circumference, body mass index (BMI), and total cholesterol (TC) and TG levels which were higher and the % of participants who consumed alcohol and the ratio of apolipoprotein (Apo) A1 to ApoB were lower in Jing ethnic minority than in Han nationality (*P* < 0.01–0.001). However, no such difference in the levels of HDL cholesterol, LDL cholesterol, ApoA1 and ApoB between the two populations (*P* > 0.05 for all).

### Genotyping

**Figure 1:**
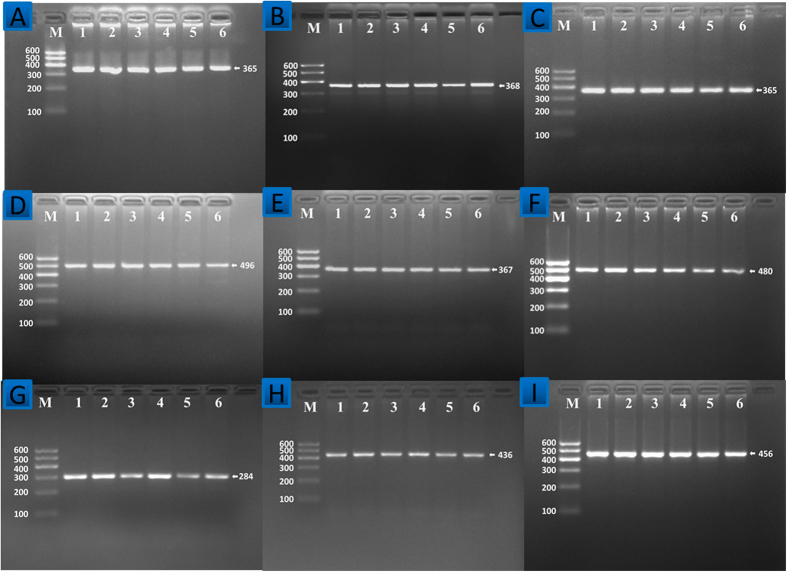
Agarose gel electrophoresis (2%) of PCR products of the *DOCK7*, *PCSK9* and *GALNT2* SNPs. Lane M: DNA ladder 100bp; PCR amplicon of (**A**) *DOCK7* rs1168013, (**B**) *DOCK7* rs10889332, (**C**) *PCSK9* rs615563, (**D**) *PCSK9* rs7552841, (**E**) *PCSK9* rs11206517, (F) *GALNT2* rs1997947, (**G**) *GALNT2* rs2760537, (**H**) *GALNT2* rs4846913 and (**I**) *GALNT2* rs11122316 SNPs were 365-, 368-, 365-, 496-, 367-, 480-, 284-, 436- and 456-bp nucleotide sequences; respectively.

**Figure 2:**
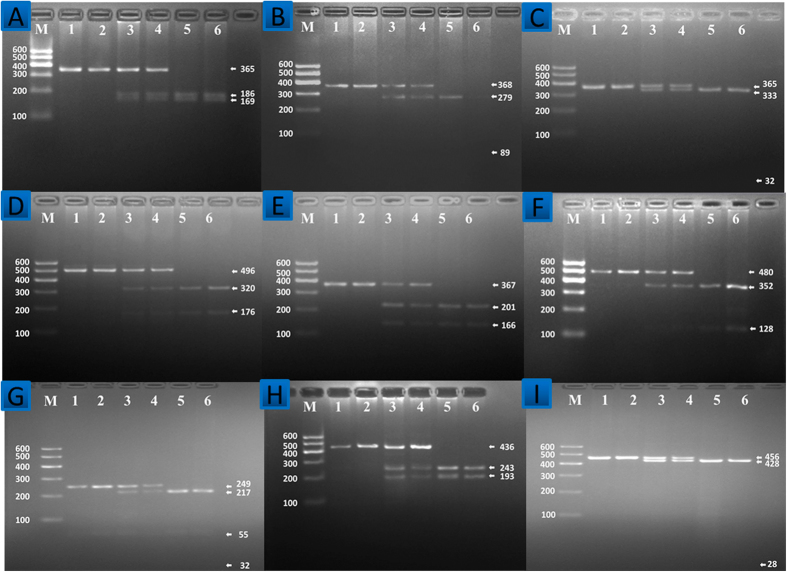
Agarose gel electrophoresis (2%) of genotyping of the *DOCK7*, *PCSK9* and *GALNT2* SNPs. Lane M: DNA ladder100bp. The genotypes of 9 SNPs were as follow: (**A**) *DOCK7* rs1168013: CC (Lanes 1 and 2, 365-bp); CG (lanes 3 and 4, 365-, 186- and 169-bp); and GG genotype (lanes 5 and 6, 186- and 169-bp). (**B**) *DOCK7* rs10889332: TT (lanes 1 and 2, 368-bp); CT (lanes 3 and 4, 368-, 279- and 89-bp); and CC genotype (lanes 5 and 6, 279- and 89-bp). (**C**) *PCSK9* rs615563: AA (lanes 1 and 2, 365-bp); AG (lanes 3 and 4, 365-, 333-, 32-bp); and GG genotype (lanes 5 and 6, 333- and 32-bp). (**D**) *PCSK9* rs7552841: TT (lanes 1 and 2, 496-bp); CT (lanes 3 and 4, 496-, 320- and 176-bp); and CC genotype (lanes 5 and 6, 320- and 176-bp). (**E**) *PCSK9* rs11206517: TT (lanes 1 and 2, 367-bp); GT (lanes 3 and 4, 367-, 201- and 166-bp); and GG (lanes 5 and 6, 201- and 166-bp). (**F**) *GALNT2* rs1997947: AA (lanes 1 and 2, 480-bp); AG (lanes 3 and 4, 480-, 352- and 128-bp); and GG genotype (lanes 5 and 6, 352- and 128-bp). (**G**) *GALNT2* rs2760537: TT (lanes 1 and 2, 249- and 55-bp); CT (lanes 3 and 4, 249-, 217-, 55- and 32-bp); and CC genotype (lanes 5 and 6, 217-, 55- and 32-bp). (**H**) *GALNT2* rs4846913: AA (lanes 1 and 2, 436-bp); AC (lanes 3 and 4, 436-, 243-and 193-bp); and CC genotype (lanes 5 and 6, 243- and 193-bp). (**I**) *GALNT2* rs11122316: AA (lanes 1 and 2, 456-bp); AG (lanes 3 and 4, 456-, 428- and 28-bp); and GG genotype (lanes 5 and 6, 428- and 28-bp). The less than 90-bp fragment was invisible in the gel owing to its fast migration speed.

Polymerase chain reaction (PCR) products of *DOCK7* rs1168013, *DOCK7* rs10889332, *PCSK9* rs615563, *PCSK9* rs7552841, *PCSK9* rs1126517, *GALNT2* rs1997947, *GALNT2* rs2760537, *GALNT2* rs4846913 and *GALNT2* rs11122316 SNPs were 365-, 368-, 365-, 496-, 367-, 480-, 284-, 436- and 456-bp nucleotide sequences after electrophoresis; respectively (Fig. 1). After restriction fragment length polymorphism (RFLP) reaction and then imaged by 2% agarose gel electrophoresis, the genotypes of the SNPs identified were labeled according to the presence and absence of the enzyme restriction sites (Fig. 2).

### Results of sequencing

**Figure 3:**
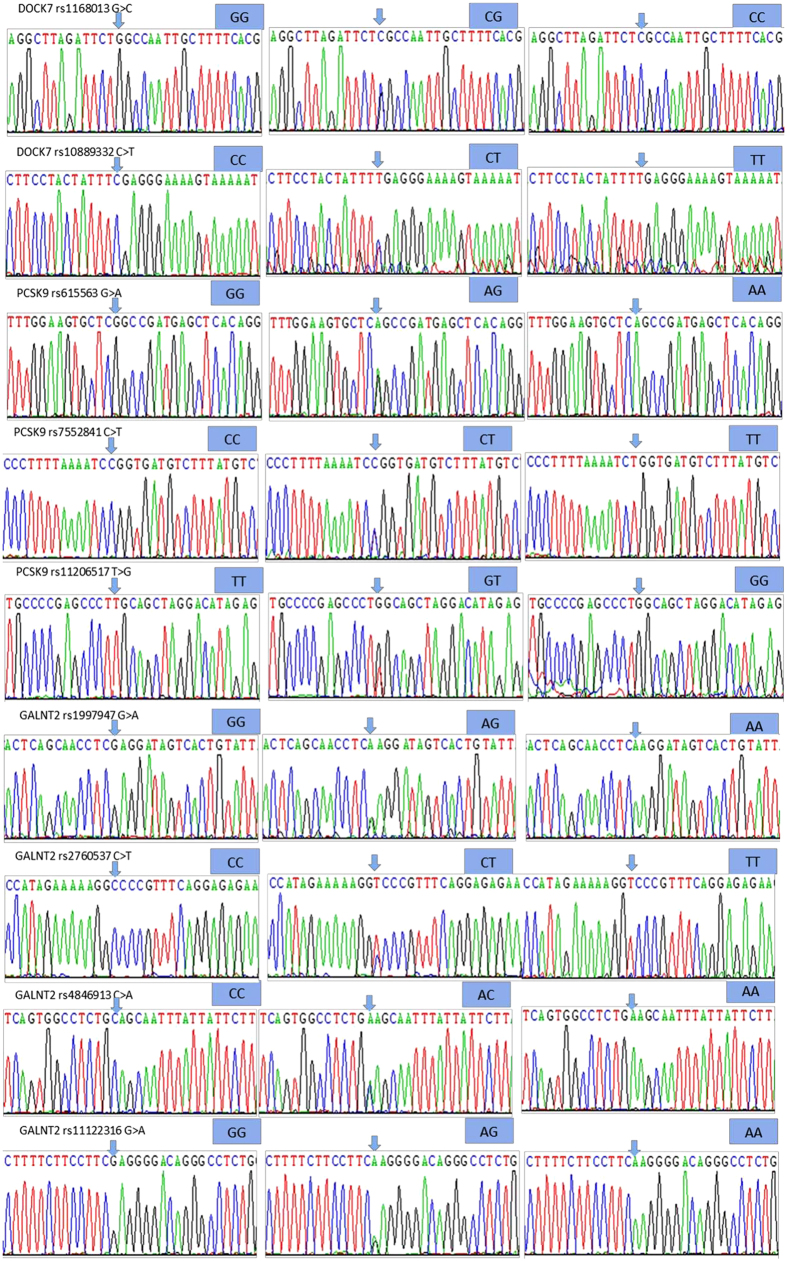
The parts of the nucleotide direct sequencing results of the *DOCK7*, *PCSK9* and *GALNT2* SNPs. DOCK7: dedicator of cytokinesis 7, PCSK9: proprotein convertase subtilisin/kexin type 9 and GALNT2: polypeptide N-acetylgalactosaminyltransferase 2.

The genotypes shown in Fig 2 by PCR-RFLP, the genotypes were also confirmed by the nucleotide direct sequencing (Fig. 3); respectively.

### Allelic and genotypic frequencies

**Figure 4:**
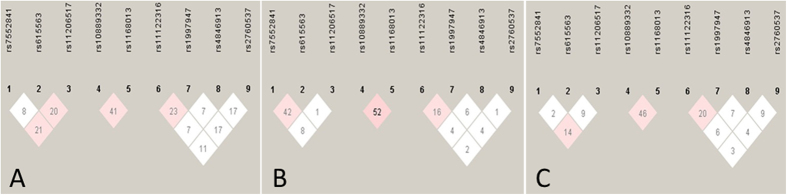
The linkage disequilibrium (LD) of the *DOCK7*, *PCSK9* and *GALNT2* SNPs. LD among the (1) *PCSK9* rs7552841, (2)*PCSK9* rs615563, (3) *PCSK9* rs11206517, (4)*DOCK7* rs10889332, (5) *DOCK7* rs1168013, (6) *GALNT2* rs11122316, (7) *GALNT2* rs1997947, (8) *GALNT2* rs4846913 and (9) *GALNT2* rs2760537 SNPs in the Jing (A), Han (B) and combined Jing and Han populations (C). The LD status is expounded by the *r*^2^ value.

Tables 2 and 3 describe the allelic and genotypic frequencies of the detected SNPs which were different between the two ethnic groups (*P* < 0.05 for all). All of the detected SNPs were in the Hardy-Weinberg equilibrium (*P* > 0.05) except *DOCK7* rs10889332 (*P* < 0.05). Linkage disequilibria (LD) were found between *PCSK9* rs6165563 and *PCSK9* rs11206517, *PCSK9* rs7552841 and *PCSK9* rs11206517, *DOCK7* rs1168013 and *DOCK7* rs10889332 and *GALNT2* rs11122316 and *GALNT2* rs1997947 in Jing, and *PCSK9* rs7552841 and *PCSK9* rs615563, *DOCK7* rs1168013 and *DOCK7* rs10889332 and *GALNT2* rs11122316 and *GALNT2* rs1997947 in Han (*P* < 0.01 for all; Fig. 4).

### Haplotype frequencies

The haplotype frequencies are listed in Table 4. The commonest haplotype was G-C-G-C-T-G-C-C-G (in the order of *DOCK7* rs1168013, *DOCK7* rs10889332, *PCSK9* rs615563, *PCSK9* rs7552841, *PCSK9* rs11206517, *GALNT2* rs1997947, *GLANT2* rs2760537, *GLANT2* rs4846913 and *GLANT2* rs11122316; >10% of the samples). The frequencies of the C-C-G-C-T-G-T-C-G, G-C-A-C-T-G-C-C-G, G-C-G-C-T-A-C-C-A, G-C-G-C-T-G-C-C-A, and G-C-G-C-T-G-T-C-A haplotypes were also different between the Jing and Han populations (*P* < 0.05 − 0.001).

### Genotypes and lipid parameters

Table 5 shows that the levels of TG (*DOCK7* rs10889332, *PCSK9* rs615563, *PCSK9* rs7552841, *GALNT2* rs1997947, *GALNT2* rs2760537, *GALNT2* rs4846913 and *GALNT2* rs11122316), HDL cholesterol (*GALNT2* rs1997947), LDL cholesterol (*DOCK7* rs1168013 and *PCSK9* rs7552841), ApoA1 (*GALNT2* rs1997947), ApoB (*DOCK7* rs10889332 and *PCSK9* rs7552841) and the ratio of ApoA1 to ApoB (*PCSK9* rs7552841) in the Jing ethnic minority were different among the three genotypes (*P* < 0.005–0.001), whereas the levels of TG (*DOCK7* rs10889332, *PCSK9* rs615563, *PCSK9* rs7552841, *PCSK9* rs11206517, *GALNT2* rs1997947, *GALNT2* rs4846913 and *GALNT2* rs11122316), HDL cholesterol (*PCSK9* rs11206517 and *GALNT2* rs4846913), LDL cholesterol (*DOCK7* rs1168013), ApoA1 (*PCSK9* rs11206517 and *GALNT2* rs4846913), ApoB (*PCSK9* rs7552841) and the ratio of ApoA1 to ApoB (*GALNT2* rs4846913) in the Han nationality were different among the genotypes (*P* < 0.005–0.001). When both minor homozygous and heterozygous were combined to enhance the power, the levels of TC (*DOCK7* rs1168013 and *PCSK9* rs7552841), TG (*DOCK7* rs10889332, *PCSK9* rs7552841, *PCSK9* rs11206517, *GALNT2* rs1997947, *GALNT2* rs2760537, *GALNT2* rs4846913 and *GALNT2* rs11122316), HDL cholesterol (*PCSK9* rs11206517, *GALNT2* rs1997947), LDL cholesterol (*DOCK7* rs1168013 and *PCSK9* rs7552841), ApoA1 (*GALNT2* rs1997947), ApoB (*DOCK7* rs10889332 and *PCSK9* rs7552841) and the ratio of ApoA1 to ApoB (*PCSK9* rs7552841) in the Jing ethnic minority were found to be different between the two genotypes (*P* < 0.005–0.001); whereas the levels of TG (*PCSK9* rs615563 and *GALNT2* rs4846913), HDL cholesterol (*PCSK9* rs11206517, *GALNT2* rs1997947 and *GALNT2* rs4846913), ApoA1 (*PCSK9* rs11206517, *GALNT2* rs1997947 and *GALNT2* rs4846913) and the ratio of ApoA1 to ApoB (*DOCK7* rs10889332, *GALNT2* rs1997947 and *GALNT2* rs4846913) in the Han nationality were different between the genotypes (*P* < 0.005–0.001).

### Haplotypes and lipid profiles

The correlation of the haplotypes and lipid profiles is shown in Table 6. Rare Hap (frequency < 3%) in both Jing and Han populations has been dropped. The carriers of C-C-G-C-T-G-T-C-G haplotype had lower TG and higher HDL cholesterol levels in Jing plus Han populations and lower TG levels in Jing population than the non-carriers of C-C-G-C-T-G-T-C-G haplotype (*P* < 0.05). There were no differences in lipid parameters between the carriers and non-carriers of C-C-G-C-T-G-T-C-G haplotype in the Han population. Haplotype G-C-A-C-T-G-C-C-G carriers had lower serum TG in the Han populations than the haplotype non-carriers (*P* < 0.05). Haplotype G-C-G-C-T-A-C-C-A carriers had higher serum TG and lower ApoA1 levels in Jing plus Han population, and higher serum TG and lower HDL cholesterol and ApoA1 in Jing population than the haplotype G-C-G-C-T-A-C-C-A non-carriers (*P* < 0.05 for each). Haplotype G-C-G-C-T-G-C-C-A carriers had lower TC, TG, LDL cholesterol and ApoB levels in Jing plus Han population, lower TC, TG and ApoB in Jing ethnic minority and lower TG levels than the haplotype G-C-G-C-T-A-C-C-A non-carriers (*P* < 0.05 for all).

### Correlation between lipid parameters and alleles or genotypes

Table 7 depicts the direction and magnitude of associations between lipid parameters and alleles or genotypes of the 9 SNPs in the Jing and Han populations. Adjusting for age, sex, BMI, smoking status, alcohol use, and exercise, logistic regression analysis showed that several the examined SNPs were significant correlated with lipid parameters.

## Discussion

In the present study, we showed for the first time the association of the *DOCK7* (rs1168013 and rs10889332), *PCSK9* (rs615563, rs7552841 and rs1126517) and *GALNT2* (rs1997947, rs2760537, rs4846913 and rs11122316) SNPs and some serum lipid parameters; the LD status and the haplotype frequencies of the detected SNPs. In addition, we also successfully replicated the association of *DOCK7* rs10889332, *PCSK9* rs615563, *PCSK9* rs7552841, *GALNT2* rs1997947, *GALNT2* rs2760537, *GALNT2* rs4846913 and *GALNT2* rs11122316 SNPs with the levels of serum TG in the Jing ethnic minority; and *DOCK7* rs10889332, *PCSK9* rs615563, *PCSK9* rs7552841, *PCSK9* rs11206517, *GALNT2* rs1997947, *GALNT2* rs4846913 and *GALNT2* rs11122316 with serum TG levels in the Han nationality.

The SNPs of rs636523 and rs12130333^22^ near the *DOCK7*/ *ANGPTL3, PCSK9* rs505151^23^ and *GALNT2* rs4846914^24^,^25^ have been associated with TG in some previous studies, but the association of the 9 SNPs and other serum lipid parameters has not been reported previously. The genotype and allele frequencies of several SNPs in this study were also not reported previously in different racial/ethnic groups. In the present study, we revealed that the genotypic and allelic frequencies of the *DOCK7* rs1168013, *DOCK7* rs10889332, *PCSK9* rs615563, *PCSK9* rs7552841, *PCSK9* rs1126517, *GALNT2* rs1997947, *GALNT2* rs2760537, *GALNT2* rs4846913 and *GALNT2* rs11122316 SNPs were different between the two ethnic groups. All of the detected SNPs were in the Hardy-Weinberg equilibrium except *DOCK7* rs10889332. The minor allele or rare homozygote genotype frequencies of the 9 SNPs in the Han nationality were in close proximity to those of CHB from the international haplotype map (HapMap; http://hapmap.ncbi.nlm.nih.gov/cgi-perl/gbrowse/hapmap24_B36/) data. The minor allele or rare homozygote genotype frequencies of the 9 detected SNPs were also lower in European ancestries than in Asian nationalities from the data. These results suggest that the prevalence of the minor allele or rare homozygote genotype frequencies of the 9 SNPs may have a racial/ethnic-specificity.

In the present reasearch, our findings also showed that there may be a racial/ethnic specific association of the 9 SNPs and lipid parameters. The association of other SNPs near *DOCK7, PCSK9* and *GALNT2* and lipid profiles has been reported previously. Through fine-mapping, previous study discovered the SNP with significant associations, with consistent effect on TG levels across ancestral groups: rs636523 near *DOCK7/ANGPTL3*. African LD patterns did not assist in narrowing association signals^22^. *PCSK9* (TG, HDL cholesterol, ApoB and ApoA1/ApoB) was shown interactions with overweight/obesity to influence serum lipid levels^23^. Our team reported that the correlations of both *GALNT2* rs2144300 and *GALNT2* rs4846914 SNPs and lipid parameters were different between the two ethnic groups^19^. However, Several GWASs and candidate gene researches failed to find the association between the *GALNT2* polymorphisms and lipid parameters^26^,^27^,^28^. There was no any effect of the *GALNT2* rs4846914 on the levels of serum TC or TG reported by Polgár *et al.* previously^26^. In Whitehall II, there was a significant correlation of the *GALNT2* variants and serum lipoprotein (a) levels. Whereas any of these findings did not confirmed in the previously meta-analysis of six studies^28^. It could be due to the effects of these SNPs were modest on serum lipid concentrations and/or lower statistical power to determine the correlation was present^26^,^29^. In addition, gene-environmental and environmental- environmental factors on lipid parameters remain to be interpreted.

Many GWASs have reported that the association of other variants near *DOCK7*, *PCSK9* and *GALNT2* and serum lipid levels is still controversial. Pleiotropic effects on the lipid profile, the potential correspondence was detected for *ANGPTL3*^30^ and *DOCK7*^31^ being highly associated with cholesterol and LDL cholesterol levels. Loss-of-function mutations in the *ANGPTL3* were associated with decreased levels of LDL cholesterol, HDL cholesterol and TG^32^. The associations observed for the *DOCK7* locus, which is involved in neurogenesis, myelination and axon formation^33^ but not in lipid metabolism probably reflect the co-localization of this gene with *ANGPTL3*. As expected, rare variants that contribute to population differences tend to be population specific, exemplified by multiple African-specific variants in *PCSK9* associated with LDL cholesterol^34^. The SNPs in intron 1 of a GalNac transferase (GALNT2) were identified as a novel lipid-associated region from GWAS and subsequent knock-down and overexpression of this gene in mouse liver clearly demonstrated that *GALNT2* can influence HDL cholesterol levels^35^.

The cause of the contradictions in correlation of the detected SNPs with lipid parameters among the different population is not completely understood. This could be because of the differences in genetic background in some degree. Compared to the Han nationality, the Jing ethnic minority had higher the value of weight, BMI, waist circumference, the serum TC and TG levels and the lower percentage of participants who consumed alcohol and the ratio of ApoA1 to ApoB. Among 56 nationalities in China, Han nationality is the largest one. Jing ethnic minority was less population nationality with the population of 22517 according to the China’s fifth national census in 2000. Approximately 90% of the Jing people live in the three islands of Wanwei, Wutou and Shanxin in the Dongxing city, Guangxi, China. About 1511, their ancestors emigrated from Vietnam to China and first settled on the aforementioned three islands. Therefore, some hereditary background and alleles/genotypes of lipid metabolism-related genes in Jing ethnic minority might be somewhat different from those in Han nationality.

Another reason could be because of the ethnic difference in their LD pattern. In this research, we detected that the frequencies of the C-C-G-C-T-G-T-C-G, G-C-A-C-T-G-C-C-G, G-C-G-C-T-A-C-C-A, G-C-G-C-T-G-C-C-A, G-C-G-C-T-G-T-C-A haplotypes were significantly different between the Jing and Han populations. The haplotypes with nine SNPs could explain much more serum lipid variation than any single SNP alone, especially for TG. Therefore, ethnic differences in the LD pattern could partially explain the discrepancy in the correlation of the detected SNPs with lipid parameters among diverse nationalities.

Several environmental factors independently such as hypertension, obesity, physical activity, dietary patterns and lifestyle are related with lipid parameters strongly^36^,^37^,^38^,^39^,^40^,^41^,^42^. There was association of gender, age, BMI, cigarette smoking, alcohol consumption, blood pressure and lipid levels in both Jing and Han populations. These detected data determined some environmental factors play an important role in determining lipid parameters. For approximately half a century it has been acknowledged that diets of high-fat particularly contain the large quantities of saturated fatty acids raise predispose individuals to hyperlipidemia and CVD^43^. In the current study, we found that the % of participants who consumed alcohol were lower in Jing than in Han nationality. Although effects of the alcohol consumption on lipid parameters appear to vary by types of specific patient or the alcohol intake patterns, and perhaps by sex and population, the subject research has been the focus of so much current studies^44^,^45^,^46^.

GWASs have identified many loci that will harbor genes relevant to the biology of lipid levels. The results show that significant associations can be identified by studying a relatively small number of subjects with extreme values of a quantitative lipoprotein trait. Re-sequencing genes at GWAS loci may reveal new rare loss-of-function mutations, creating potential new therapeutic targets for decreasing the prevalence of heart disease. Until recently, most genome-wide efforts have used genotyping arrays and imputation to assay most of the common variation across the genome. Recent technological advances have enabled whole genome sequencing approaches, which hold the promise of discovery of novel rare variants with large effects on lipid levels and heart disease risk.

There are several potential limitations in the present study. Firstly, the sample size is a bit small as compared with many previous GWASs. Hence, further studies with larger sample sizes are needed to confirm our results. Secondly, interactions of gene-environmental or environmental-environmental factors on serum lipid traits remain to be detected. Thirdly, there are no independent haplotypes of each gene considered instead. Phasing of the 9 SNPs is so far away on chromosome 1 (~40MB). There will be many recombination events that will be missed due to the lack of information across the region. Finally, although we have detected the effects of 9 SNPs in the *PCSK9*, *DOCK7* and *GALNT2* on serum lipid levels in this study, there are still many lipid-related SNPs and the interactions of SNP-SNP and/or SNP-environmental factors. What’s more, the relevance of this finding has to be defined in further high caliber of studies including incorporating the genetic information of the *DOCK7*, *PCSK9* and *GALNT2* SNPs and their haplotypes and *in vitro* functional studies to confirm the impact of a variant on a molecular level.

In summary, several SNPs in the present study were associated with TG (rs10889332, rs615563, rs7552841, rs1997947, rs2760537, rs4846913 and rs11122316), HDL cholesterol (rs1997947), LDL cholesterol (rs1168013 and rs7552841), ApoA1 (rs1997947), ApoB (rs10889332 and rs7552841), and the ApoA1/ApoB ratio (rs7552841) in the Jing population, whereas they were associated with TG (rs10889332, rs615563, rs7552841, rs11206517, rs1997947, rs4846913 and rs11122316), HDL cholesterol (rs11206517 and rs4846913), LDL cholesterol (rs1168013), ApoA1 (rs11206517 and rs4846913), ApoB (rs7552841), and the ApoA1/ApoB ratio (rs4846913) in the Han participants. The frequencies of several haplotypes among the 9 SNPs were also different between the Jing and Han populations. The differences in lipid parameters between the Jing and Han populations might result from different SNPs and their haplotypes partially.

## Materials and methods

### Subjects and research design

For the current study, 881 unrelated subjects (456 males, 51.76% and 425 females, 48.24%) of Jing and 988 (536 men, 54.25% and 452 women, 45.75%) unrelated individuals of Han from Dongxing city, Guangxi Zhuang Autonomous Region, China were selected randomly from our randomized, stratified samples. The subjects’ age ranged from 15 to 80 years and with the average age of 56.69 ± 13.39 years in Jing and 56.18 ± 12.85 years in Han. All participants were healthy with no disease history of atherosclerosis, CVD, diabetes, thyroid and/or kidney. When blood samples were taken, none of them used lipid-modulating therapy such as fibrates or statins. The study design was approved by the Ethics Committee of the First Affiliated Hospital, Guangxi Medical University. Written informed consent was obtained from all participants. All experiments were performed in accordance with relevant guidelines and regulations^47^,^48^,^49^,^50^.

## Data collection

### Epidemiological investig**a**tion and measurements of biochemical markers

Participants participated in baseline examination conducted in the study center by trained staff following standardized protocols, which included anthropometric, blood pressure measurements, height, weight (without shoes) and waist circumference parameters (in cm was measured at the midpoint between the lower ribs and the iliac crest), a blood sample collection as well as a personal interview on medical history, a sociodemographic, socioeconomic status and lifestyle questionnaire and a self-administered food frequency questionnaire; BMI was calculated as the ratio of weight (kg) to squared height (m^2^). During the blood sample collection, 5 ml of venous blood were drawn, rapidly processed and serum lipids, lipoproteins, and apolipoproteins were measured by enzymatic methods with commercially available kits: RANDOX Laboratories Ltd., Ardmore, Diamond Road, Crumlin Co. Antrim, United Kingdom, BT29 4QY and Daiichi Pure Chemicals Co., Ltd., Tokyo, Japan, and the immunoturbidimetric assay using a commercial kit: RANDOX Laboratories Ltd. in the Clinical Science Experiment Center of the First Affiliated Hospital, Guangxi Medical University with an autoanalyzer: Type 7170A; Hitachi Ltd., Tokyo, Japan. The normal values of serum lipid phenotypes in our Clinical Science Experiment Center were as follows: TC, 3.10–5.17 mmol/L; TG, 0.56–1.70 mmol/L; HDL cholesterol, 1.16–1.42 mmol/L; LDL cholesterol, 2.70–3.10 mmol/L; ApoA1, 1.20–1.60 g/L; ApoB, 0.80–1.05 g/L; and the ratio of ApoA1 to ApoB, 1.00–2.50; respectively^50^.

### SNPs selection

Nine TG-related loci in the *DOCK7*, *PCSK9* and *GALNT2* were selected by three criteria encompass (i) Tag SNPs, which were established by Haploview (Broad Institute of MIT and Harvard, USA, version 4.2) and functional/missense SNPs in functional area of the gene fragments (http://www.ncbi.nlm.nih.gov/SNP/snp); (ii) a known minor allele frequency higher than 1% in European ancestry from the Human Genome Project Database; and (iii) the target SNP region should be adequately replicated by PCR, and the polymorphic site should have a commercially available restriction endonuclease enzyme cleavage site to be genotyped with RFLP.

### Genotyping

DNA was isolated from blood samples using DNA Blood Midi kits (Qiagen, Hilden, Germany) following the protocol recommended by the vendor. We identified 9 SNPs genotyping by PCR-RFLP. The characteristics of each SNP and the details of each primer pair, annealing temperature, length of the PCR products and corresponding restriction enzyme used for genotyping are summarized in supplemental Tables 1 and 2. The PCR products of the samples (two samples of each genotype) were sequenced with an ABI Prism 3100 (Applied Biosystems, international Equipment Trading Ltd., Vernon Hill, IL, USA) in Shanghai Sangon Biological Engineering Technology & Services Co., Ltd., China.

### Statistical methods

The statistical analyses were done with the statistical software package SPSS 17.0 (SPSS Inc., Chicago, Illinois). Data were presented as the mean ± SD for those, that are normally distributed, and the medians and interquartile ranges for TG, which is not normally distributed. Clinical characteristics between the Jing and Han populations were compared by Student’s unpaired *t*-test. The allele, genotype and haplotype distribution between the Jing and Han populations were analyzed by the chi-squared test; and the standard goodness-of-fit verified the test Hardy-Weinberg equilibrium. Haploview (Broad Institute of MIT and Harvard, USA, version 4.2) analyzed the haplotype frequencies and pair-wise LD among the detected SNPs. The correlation of genotypes and lipid profiles was calculated by ANCOVA. Any SNPs associated with the lipid profiles at the value of *P* < 0.005 (corresponding to *P* < 0.05 after adjusting for 9 independent tests by the Bonferroni correction) were considered statistically significant. Unconditional logistic regression was used to assess the assocation between lipid parameters and genotypes (common homozygote genotype = 1, heterozygote genotype = 2, rare homozygote genotype = 3) or alleles (the minor allele non-carrier = 1, the minor allele carrier = 2). Gender, age, BMI, alcohol consumption, cigarette smoking and hypertension were adjusted for statistical analysis. Two-sided *P* value of less than 0.05 was considered statistically significant.

## Additional Information

**How to cite this article**: Guo, T. *et al.* Association between the DOCK7, PCSK9 and GALNT2 Gene Polymorphisms and Serum Lipid levels. *Sci. Rep.* **6**, 19079; doi: 10.1038/srep19079 (2016).

## Supplementary Material

Supplemental Tables 1 and 2

## Figures and Tables

**Table 1:** Lipid profiles and clinical characteristics in the two ethnic groups.

Characteristics	Jing	Han	*test-statistic*	*P-value*
Number (n)	881	988		
Gender (Male/Female)	456/425	536/452	1.161	0.281
Age (years)	56.69 ± 13.39^1^	56.18 ± 12.85	−0.726	0.468
Height (cm)	156.51 ± 7.67	156.03 ± 7.79	1.133	0.257
Weight (kg)	57.62 ± 9.86	55.66 ± 9.37	3.768	0.000
Body mass index (kg/m^2^)	23.46 ± 3.25	22.82 ± 3.23	3.642	0.000
Waist circumference (cm)	79.98 ± 9.04	77.49 ± 8.94	5.115	0.000
SBP (mmHg)	131.65 ± 22.03	132.66 ± 41.95	−0.550	0.582
DBP (mmHg)	80.37 ± 10.54	80.84 ± 10.17	−0.848	0.397
Pulse pressure (mmHg)	51.28 ± 17.54	51.82 ± 10.15	−0.315	0.753
Cigarette smoking [n (%)]				
Nonsmoker	775 (87.9)	846 (85.6)		
≤20 Cigarettes/day	27 (3.1)	36 (3.6)	1.634	0.442
>20 Cigarettes/day	79 (9.0)	106 (10.8)		
Alcohol consumption [n (%)]				
Nondrinker	788 (89.4)	834 (84.4)		
≤25 g/day	50 (5.7)	35 (3.5)	25.016	0.000
>25 g/day	43 (4.9)	119 (12.1)		
Blood glucose level (mmol/L)	6.71 ± 1.71	6.63 ± 1.08	0.983	0.326
Total cholesterol (mmol/L)	5.13 ± 0.93	4.89 ± 0.87	4.834	0.000
Triglyceride (mmol/L)	1.41 (1.12)^2^	1.32 (1.09)	−2.890	0.004
HDL cholesterol (mmol/L)	1.79 ± 0.52	1.80 ± 0.45	−0.231	0.817
LDL cholesterol (mmol/L)	2.85 ± 0.44	2.82 ± 0.44	1.314	0.189
Apolipoprotein (Apo) A1 (g/L)	1.30 ± 0.23	1.32 ± 0.20	−1.592	0.112
ApoB (g/L)	1.06 ± 0.25	1.03 ± 0.24	1.837	0.066
ApoA1/ApoB	1.30 ± 0.38	1.35 ± 0.37	−2.465	0.014

SBP, systolic blood pressure; DBP, diastolic blood pressure; HDL, high density lipoprotein; LDL, low density lipoprotein;

^1^Mean ± SD determined by *t*-test.

^2^Median (interquartile range) tested by the Wilcoxon-Mann-Whitney test.

**Table 2:** Prevalence of genotype frequencies in the different populations [n (%)].

SNP	Genotype	Jing (n = 881)	Han (n = 988)	X^2^	*P*-value
*DOCK7* rs1168013 G > C	GG	367 (41.65)	482 (48.75)	7.457	0.024
	CG	409 (46.40)	413 (41.81)		
	CC	105 (11.95)	93 (9.44)		
	HWE (P)	0.581	0.739		
*DOCK7* rs10889332 C > T	CC	447 (50.69)	575 (58.19)	10.851	0.004
	CT	341 (38.74)	347 (35.14)		
	TT	93 (10.57)	66 (6.67)		
	HWE (P)	0.023	0.168		
*PCSK9* rs615563 G > A	GG	536 (60.80)	664 (67.22)	6.223	0.045
	AG	294 (33.38)	279 (28.20)		
	AA	51 (5.82)	45 (4.58)		
	HWE (P)	0.208	0.271		
*PCSK9* rs7552841 C > T	CC	571 (64.78)	689 (69.72)	6.063	0.048
	CT	264 (30.01)	269 (27.22)		
	TT	46 (5.21)	30 (3.06)		
	HWE (P)	0.359	0.549		
*PCSK9* rs11206517 T > G	TT	723 (82.08)	859 (86.95)	6.357	0.042
	GT	146 (16.54)	121 (12.22)		
	GG	12(1.38)	8 (0.83)		
	HWE (P)	0.142	0.108		
*GALNT2* rs1997947 G > A	GG	529 (60.03)	661 (66.94)	7.586	0.023
	AG	297 (33.69)	283 (28.61)		
	AA	55 (6.28)	44 (4.45)		
	HWE (P)	0.129	0.056		
*GALNT2* rs2760537 C > T	CC	344 (39.05)	446 (45.14)	6.291	0.043
	CT	406 (46.10)	427 (43.19)		
	TT	131 (14.85)	115 (11.67)		
	HWE (P)	0.531	0.408		
*GALNT2* rs4846913 C > A	CC	554 (62.94)	685 (69.30)	6.292	0.043
	AC	281 (31.85)	263 (26.67)		
	AA	46 (5.21)	40 (4.03)		
	HWE (P)	0.188	0.232		
*GALNT2* rs11122316 G > A	GG	320 (36.29)	410 (41.53)	6.541	0.038
	AG	429 (48.70)	468 (47.36)		
	AA	132 (15.01)	110 (11.11)		
	HWE (P)	0.546	0.171		

SNP: single nucleotide polymorphism; HDL, high density lipoprotein; LDL, low density lipoprotein; HWE, Hardy-Weinberg equilibrium; DOCK7: Dedicator of cytokinesis 7; PCSK9: Proprotein convertase subtilisin/kexin type 9; GALNT2: N-acetylgalactosaminyltransferase 2.

**Table 3:** Prevalence of allele frequencies in the different populations [n (%)].

SNP	Allele	Jing (n = 881)	Han (n = 988)	X^2^	P-value
*DOCK7* rs1168013	G/C	1143 (64.85)/619 (35.15)	1376 (69.65)/600 (30.35)	7.173	0.007
*DOCK7* rs10889332	C/T	1234 (70.06)/528 (29.94)	1497 (75.76)/479 (24.24)	11.313	0.001
*PCSK9* rs615563	G/A	1365 (77.49)/397 (22.51)	1607 (81.32)/369 (18.68)	6.167	0.013
*PCSK9* rs7552841	C/T	1406 (79.79)/356 (20.21)	1647 (83.33)/329 (16.67)	5.752	0.016
*PCSK9* rs11206517	T/G	1592 (90.35)/170 (9.65)	1839 (93.06)/137 (6.94)	6.627	0.010
*GALNT2* rs1997947	G/A	1355 (76.88)/407 (23.12)	1606 (81.25)/370 (18.75)	7.945	0.005
*GALNT2* rs2760537	C/T	1094 (62.10)/668 (37.90)	1319 (66.74)/657 (33.26)	6.437	0.011
*GALNT2* rs4846913	C/A	1390 (78.87)/372 (21.13)	1633 (82.64)/343 (17.36)	6.293	0.012
*GALNT2* rs11122316	G/A	1068 (60.64)/694 (39.36)	1289 (65.21)/687 (34.79)	6.126	0.013

SNP: single nucleotide polymorphism; DOCK7: Dedicator of cytokinesis 7; PCSK9: Proprotein convertase subtilisin/kexin type 9; GALNT2: N-acetylgalactosaminyltransferase 2.

**Table 4:** Frequencies of haplotypes among 9 SNPs of the *DOCK7, PCSK9* and *GALNT2* genes in the two ethnic groups [n (%)].

Haplotype									Jing	Han	X^2^	*P*-value
A	B	C	D	E	F	G	H	I				
C	C	G	C	T	G	C	C	G	61 (3.1)	47 (2.7)	0.011	0.916
C	C	G	C	T	G	T	C	G	5 (0.2)	59 (3.3)	63.522	1.66 × 10^−15^
G	C	A	C	T	G	C	C	G	92 (4.7)	47 (2.7)	6.786	0.009
G	C	G	C	T	A	C	C	A	4 (0.2)	55 (3.1)	60.831	6.52 × 10^−15^
G	C	G	C	T	G	C	C	A	133 (6.8)	66 (3.7)	11.968	0.001
G	C	G	C	T	G	C	C	G	262 (13.3)	187 (10.6)	1.908	0.167
G	C	G	C	T	G	T	C	A	80 (4.1)	42 (2.4)	5.642	0.018
G	C	G	C	T	G	T	C	G	105 (5.3)	86 (4.9)	0.088	0.767
Rare Hap (frequency <3%) in both Jing & Han populations has been dropped												

A, *DOCK7* rs1168013; B, *DOCK7* rs10889332; C, *PCSK9* rs615563; D, *PCSK9* rs7552841; E, *PCSK9* rs11206517; F, *GALNT2* rs1997947; G, *GLANT2* rs2760537; H, *GLANT2* rs4846913; I, *GLANT2* rs11122316; DOCK7, Dedicator of cytokinesis 7; PCSK9, Proprotein convertase subtilisin/kexin type 9; GALNT2, N-acetylgalactosaminyltransferase 2.

**Table 5:** Lipid profiles according to genotypes for the two ethnic groups.

Genotype	n	Total cholesterol (mmol/L)	Triglyceride (mmol/L)	HDL cholesterol (mmol/L)	LDL cholesterol (mmol/L)	Apolipoprotein (Apo) A1 (g/L)	Apolipoprotein (Apo) B (g/L)	ApoA1/ ApoB
*DOCK7* **rs1168013 G > C**								
Jing								
GG	367	4.97 ± 0.92	1.40(1.10)	1.81 ± 0.47	2.67 ± 0.55	1.31 ± 0.21	1.04 ± 0.24	1.31 ± 0.37
CG	409	5.13 ± 0.94	1.41 (1.14)	1.80 ± 0.42	2.83 ± 0.42	1.30 ± 0.25	1.06 ± 0.28	1.29 ± 0.39
CC	105	5.17 ± 0.91	1.51 (1.18)	1.71 ± 0.45	2.84 ± 0.42	1.29 ± 0.19	1.07 ± 0.25	1.29 ± 0.37
*F*		4.430	3.504	1.840	6.747	0.225	3.360	1.390
*P*		0.012	0.031	0.160	0.001	0.775	0.035	0.250
Han								
GG	482	4.80 ± 0.87	1.27 (1.04)	1.86 ± 0.54	2.80 ± 0.44	1.35 ± 0.21	1.00 ± 0.24	1.37 ± 0.39
CG	413	4.95 ± 0.87	1.31 (1.06)	1.75 ± 0.51	2.87 ± 0.44	1.30 ± 0.21	1.04 ± 0.25	1.36 ± 0.36
CC	93	5.09 ± 0.72	1.60 (1.26)	1.65 ± 0.45	2.97 ± 0.41	1.30 ± 0.19	1.14 ± 0.23	1.18 ± 0.29
*F*		2.894	3.458	2.436	5.794	1.491	3.561	1.960
*P*		0.056	0.032	0.088	0.003	0.226	0.029	0.142
*DOCK7*rs10889332 C > T								
**Jing**								
CC	447	5.05 ± 0.93	1.31 (1.06)	1.83 ± 0.43	2.78 ± 0.43	1.34 ± 0.26	1.02 ± 0.22	1.34 ± 0.39
CT	341	5.15 ± 0.88	1.49 (1.20)	1.78 ± 0.45	2.83 ± 0.45	1.30 ± 0.21	1.06 ± 0.24	1.28 ± 0.37
TT	93	5.38 ± 1.02	1.91 (1.28)	1.69 ± 0.54	2.94 ± 0.46	1.29 ± 0.25	1.21 ± 0.33	1.17 ± 0.32
*F*		2.624	6.928	1.226	3.628	1.202	10.896	3.207
*P*		0.073	0.001	0.294	0.027	0.301	0.000	0.041
Han								
CC	575	4.85 ± 0.83	1.27 (1.07)	1.81 ± 0.54	2.83 ± 0.42	1.34 ± 0.20	1.02 ± 0.23	1.39 ± 0.42
CT	347	4.88 ± 0.91	1.33 (1.10)	1.79 ± 0.50	2.84 ± 0.47	1.32 ± 0.24	1.03 ± 0.26	1.34 ± 0.34
TT	66	5.27 ± 0.88	1.73 (1.34)	1.70 ± 0.60	3.02 ± 0.38	1.31 ± 0.20	1.12 ± 0.21	1.22 ± 0.34
*F*		2.271	5.660	0.364	3.109	0.879	4.625	5.033
*P*		0.104	0.004	0.695	0.045	0.416	0.010	0.007
*PCSK9*rs615563 G > A								
Jing								
GG	536	5.07 ± 0.88	1.34 (1.10)	1.87 ± 0.64	2.80 ± 0.43	1.33 ± 0.24	1.03 ± 0.23	1.33 ± 0.38
AG	294	5.18 ± 0.94	1.51 (1.21)	1.81 ± 0.42	2.83 ± 0.45	1.31 ± 0.24	1.09 ± 0.26	1.24 ± 0.37
AA	51	5.41 ± 1.18	1.58 (1.26)	1.76 ± 0.45	2.98 ± 0.46	1.29 ± 0.22	1.12 ± 0.33	1.27 ± 0.38
*F*		1.544	5.479	1.668	2.125	1.065	1.906	1.598
*P*		0.214	0.004	0.190	0.120	0.345	0.150	0.203
Han								
GG	664	4.84 ± 0.85	1.26 (1.04)	1.90 ± 0.40	2.82 ± 0.43	1.37 ± 0.19	1.02 ± 0.24	1.37 ± 0.38
AG	279	4.98 ± 0.90	1.27 (1.11)	1.78 ± 0.53	2.89 ± 0.45	1.32 ± 0.20	1.06 ± 0.26	1.32 ± 0.27
AA	45	5.05 ± 0.77	1.47 (1.15)	1.78 ± 0.52	2.95 ± 0.41	1.31 ± 0.20	1.07 ± 0.23	1.30 ± 0.37
*F*		0.825	6.574	0.533	1.105	1.101	2.041	1.674
*P*		0.439	0.001	0.587	0.332	0.333	0.131	0.188
*PCSK9* rs7552841 C > T								
Jing								
CC	571	5.02 ± 0.90	1.33 (1.06)	1.89 ± 0.52	2.76 ± 0.43	1.31 ± 0.25	1.01 ± 0.22	1.36 ± 0.39
CT	264	5.30 ± 0.93	1.56 (1.26)	1.81 ± 0.45	2.91 ± 0.44	1.30 ± 0.21	1.14 ± 0.26	1.19 ± 0.34
TT	46	5.44 ± 1.04	1.58 (1.22)	1.75 ± 0.44	2.96 ± 0.45	1.29 ± 0.27	1.18 ± 0.30	1.16 ± 0.30
*F*		3.683	8.648	1.429	6.058	0.073	17.987	12.017
*P*		0.026	0.000	0.240	0.002	0.930	0.000	0.000
Han								
CC	689	4.86 ± 0.86	1.31 (1.07)	1.89 ± 0.62	2.83 ± 0.44	1.37 ± 0.19	1.02 ± 0.24	1.36 ± 0.38
CT	269	4.90 ± 0.84	1.32 (1.10)	1.81 ± 0.59	2.85 ± 0.41	1.33 ± 0.20	1.04 ± 0.24	1.34 ± 0.36
TT	30	5.42 ± 1.12	1.78 (1.51)	1.78 ± 0.49	3.11 ± 0.59	1.31 ± 0.20	1.21 ± 0.33	1.20 ± 0.33
*F*		3.672	8.841	0.666	3.611	1.756	5.595	3.144
*P*		0.026	0.000	0.514	0.028	0.174	0.004	0.044
*PCSK9* rs11206517 T > G								
**Jing**								
TT	723	5.07 ± 0.90	1.40 (1.12)	1.83 ± 0.45	2.80 ± 0.44	1.35 ± 0.25	1.04 ± 0.23	1.31 ± 0.37
GT	146	5.36 ± 0.98	1.47 (1.10)	1.79 ± 0.44	2.89 ± 0.43	1.29 ± 0.23	1.14 ± 0.30	1.27 ± 0.41
GG	12	5.61 ± 1.16	1.75 (1.35)	1.41 ± 0.57	3.03 ± 0.57	1.27 ± 0.26	1.30 ± 0.44	1.02 ± 0.20
*F*		0.356	4.366	4.202	0.164	3.059	2.062	1.692
*P*		0.694	0.013	0.015	0.849	0.048	0.128	0.185
Han								
TT	859	4.85 ± 0.77	1.28 (1.06)	1.82 ± 0.53	2.84 ± 0.38	1.34 ± 0.19	1.03 ± 0.25	1.36 ± 0.37
GT	121	4.89 ± 0.88	1.58 (1.24)	1.59 ± 0.41	2.85 ± 0.44	1.33 ± 0.21	1.05 ± 0.23	1.34 ± 0.50
GG	8	4.95 ± 0.38	1.95 (1.26)	1.52 ± 0.42	2.91 ± 0.34	1.24 ± 0.15	1.07 ± 0.24	1.24 ± 0.34
*F*		0.254	6.780	5.430	0.229	7.519	0.172	1.116
*P*		0.775	0.001	0.005	0.795	0.001	0.842	0.328
*GALNT2* rs1997947 G > A								
Jing								
GG	529	5.07 ± 0.94	1.29 (1.06)	1.84 ± 0.44	2.71 ± 0.53	1.33 ± 0.24	1.04 ± 0.24	1.34 ± 0.37
AG	297	5.17 ± 0.84	1.55 (1.23)	1.77 ± 0.46	2.80 ± 0.44	1.29 ± 0.22	1.08 ± 0.27	1.26 ± 0.39
AA	55	5.22 ± 0.92	1.67 (1.50)	1.52 ± 0.30	2.87 ± 0.42	1.14 ± 0.13	1.09 ± 0.21	1.10 ± 0.31
*F*		0.687	5.251	5.226	1.549	9.989	0.010	3.799
*P*		0.503	0.005	0.005	0.213	0.000	0.990	0.023
Han								
GG	661	4.88 ± 0.87	1.27 (1.04)	1.81 ± 0.45	2.84 ± 0.44	1.33 ± 0.20	1.02 ± 0.25	1.37 ± 0.37
AG	283	4.90 ± 0.88	1.38 (1.16)	1.80 ± 0.55	2.86 ± 0.45	1.31 ± 0.20	1.05 ± 0.24	1.33 ± 0.39
AA	44	4.99 ± 0.69	2.16 (1.71)	1.42 ± 0.36	2.93 ± 0.29	1.20 ± 0.15	1.12 ± 0.16	1.09 ± 0.20
*F*		0.369	6.376	4.391	0.501	4.573	0.873	3.144
*P*		0.692	0.002	0.013	0.606	0.011	0.418	0.044
*GALNT2* rs2760537 C > T								
Jing								
CC	344	5.05 ± 0.95	1.36 (1.15)	1.82 ± 0.46	2.80 ± 0.42	1.31 ± 0.25	1.04 ± 0.23	1.30 ± 0.35
CT	406	5.15 ± 0.90	1.41 (1.08)	1.78 ± 0.41	2.81 ± 0.54	1.30 ± 0.22	1.06 ± 0.25	1.30 ± 0.40
TT	131	5.23 ± 0.96	1.54 (1.24)	1.76 ± 0.52	2.83 ± 0.34	1.29 ± 0.21	1.07 ± 0.28	1.29 ± 0.39
*F*		2.062	5.588	0.038	0.232	0.234	0.525	0.163
*P*		0.128	0.004	0.963	0.793	0.792	0.592	0.850
Han								
CC	446	4.86 ± 0.87	1.30 (1.03)	1.82 ± 0.55	2.83 ± 0.45	1.32 ± 0.20	1.02 ± 0.23	1.37 ± 0.38
CT	427	4.87 ± 0.84	1.31 (1.09)	1.79 ± 0.54	2.85 ± 0.41	1.32 ± 0.20	1.03 ± 0.24	1.34 ± 0.35
TT	115	5.06 ± 0.91	1.46 (1.21)	1.76 ± 0.49	2.93 ± 0.47	1.31 ± 0.21	1.07 ± 0.28	1.30 ± 0.42
*F*		0.835	4.692	1.401	0.879	0.008	0.833	0.290
*P*		0.434	0.009	0.247	0.416	0.992	0.435	0.749
*GALNT2* rs4846913 C > A								
Jing								
CC	554	5.14 ± 0.94	1.37 (1.10)	1.83 ± 0.45	2.81 ± 0.44	1.32 ± 0.24	1.05 ± 0.26	1.32 ± 0.38
AC	281	5.07 ± 0.88	1.54 (1.11)	1.82 ± 0.50	2.82 ± 0.41	1.27 ± 0.20	1.06 ± 0.23	1.27 ± 0.37
AA	46	5.30 ± 1.02	1.69 (1.49)	1.73 ± 0.43	2.86 ± 0.62	1.26 ± 0.24	1.09 ± 0.21	1.21 ± 0.34
*F*		0.378	5.388	4.764	0.406	2.828	0.207	1.254
*P*		0.685	0.005	0.009	0.667	0.060	0.813	0.286
Han								
CC	685	4.86 ± 0.88	1.25 (1.03)	1.84 ± 0.53	2.84 ± 0.44	1.34 ± 0.21	1.02 ± 0.24	1.39 ± 0.37
AC	263	4.95 ± 0.82	1.50 (1.16)	1.77 ± 0.47	2.87 ± 0.43	1.27 ± 0.17	1.05 ± 0.25	1.27 ± 0.35
AA	40	5.03 ± 0.88	1.54 (1.24)	1.65 ± 0.48	2.93 ± 0.47	1.24 ± 0.20	1.06 ± 0.24	1.24 ± 0.43
*F*		0.527	5.320	8.741	0.410	9.946	0.945	5.623
*P*		0.591	0.005	0.000	0.664	0.000	0.389	0.004
*GALNT2* rs11122316 G > A								
Jing								
GG	320	5.06 ± 0.92	1.30 (1.03)	1.87 ± 0.40	2.79 ± 0.46	1.32 ± 0.21	1.06 ± 0.24	1.32 ± 0.37
AG	429	5.17 ± 0.95	1.49 (1.16)	1.81 ± 0.52	2.84 ± 0.42	1.31 ± 0.24	1.06 ± 0.25	1.31 ± 0.37
AA	132	5.19 ± 0.95	1.54 (1.24)	1.73 ± 0.45	2.87 ± 0.43	1.28 ± 0.24	1.06 ± 0.26	1.28 ± 0.39
*F*		2.561	6.549	3.245	1.726	1.231	0.414	0.237
*P*		0.078	0.002	0.040	0.179	0.293	0.661	0.789
Han								
GG	410	4.85 ± 0.84	1.25 (1.03)	1.81 ± 0.55	2.83 ± 0.42	1.34 ± 0.21	1.02 ± 0.23	1.36 ± 0.38
AG	468	4.92 ± 0.91	1.31 (1.07)	1.78 ± 0.51	2.84 ± 0.46	1.32 ± 0.21	1.02 ± 0.26	1.35 ± 0.37
AA	110	4.97 ± 0.82	1.49 (1.32)	1.73 ± 0.48	2.93 ± 0.42	1.32 ± 0.20	1.09 ± 0.23	1.29 ± 0.35
*F*		1.172	6.503	0.902	1.414	1.041	2.670	0.920
*P*		0.310	0.002	0.406	0.244	0.354	0.070	0.399

HDL, high density lipoprotein; LDL, low density lipoprotein; The *P*-value calculated by ANCOVA, using general linear models, and adjusted for age, sex, BMI, smoking status, alcohol use, glucose and hypertension, and less than 0.005 was considered statistically significant after adjusting by Bonferroni correction.

n = sample size.

**Table 6:** Lipid profiles according to haplotypes for the two ethnic groups.

Haplotype	Group	n	Total cholesterol (mmol/L)	Triglyceride (mmol/L)	HDL cholesterol (mmol/L)	LDL cholesterol (mmol/L)	Apolipoprotein (Apo) A1 (g/L)	Apolipoprotein (Apo) B (g/L)	ApoA1/ ApoB
C-C-G-C-T-G-T-C-G	Jing plus Han	1869							
	Carrier	64	4.98 ± 0.86	1.30(1.08)	1.84 ± 0.50	2.82 ± 0.43	1.31 ± 0.23	1.03 ± 0.24	1.34 ± 0.38
	Non-carrier	1805	4.99 ± 0.90	1.39 (1.10)	1.75 ± 0.49	2.84 ± 0.43	1.31 ± 0.21	1.05 ± 0.25	1.31 ± 0.38
	*F*		0.035	−2.397	5.661	0.269	0.011	0.736	0.355
	*P*		0.852	0.017	0.017	0.604	0.916	0.391	0.552
	Jing	881							
	Carrier	5	5.11 ± 0.92	1.30 (1.06)	1.83 ± 0.44	2.81 ± 0.43	1.29 ± 0.26	1.04 ± 0.23	1.30 ± 0.39
	Non-carrier	876	5.13 ± 0.85	1.44 (1.14)	1.77 ± 0.45	2.82 ± 0.42	1.30 ± 0.22	1.06 ± 0.25	1.29 ± 0.38
	*F*		0.062	−2.414	1.672	0.077	0.232	0.665	0.008
	*P*		0.803	0.016	0.196	0.782	0.630	0.415	0.927
	Han	988							
	Carrier	59	4.85 ± 0.85	1.29 (1.09)	1.86 ± 0.55	2.83 ± 0.44	1.33 ± 0.21	1.02 ± 0.24	1.37 ± 0.39
	Non-carrier	929	4.88 ± 0.87	1.33 (1.07)	1.74 ± 0.52	2.85 ± 0.44	1.32 ± 0.20	1.04 ± 0.24	1.34 ± 0.38
	*F*		0.112	−0.916	3.735	0.069	0.550	0.001	0.097
	*P*		0.738	0.360	0.054	0.793	0.458	0.973	0.755
G-C-A-C-T-G-C-C-G	Jing plus Han	1869							
	Carrier	139	4.98 ± 0.88	1.36 (1.06)	1.79 ± 0.46	2.83 ± 0.43	1.32 ± 0.21	1.04 ± 0.24	1.33 ± 0.38
	Non-carrier	1730	5.01 ± 0.94	1.37 (1.10)	1.77 ± 0.50	2.84 ± 0.44	1.30 ± 0.22	1.05 ± 0.24	1.31 ± 0.38
	*F*		0.015	−0.926	0.112	0.009	0.925	0.011	0.387
	*P*		0.901	0.354	0.738	0.924	0.336	0.915	0.534
	Jing	881							
	Carrier	92	5.10 ± 0.89	1.41 (1.12)	1.79 ± 0.47	2.81 ± 0.43	1.30 ± 0.23	1.06 ± 0.24	1.29 ± 0.39
	Non-carrier	789	5.17 ± 0.96	1.51 (1.16)	1.78 ± 0.45	2.82 ± 0.42	1.29 ± 0.21	1.06 ± 0.25	1.28 ± 0.38
	*F*		1.009	−0.756	0.183	0.105	0.157	0.152	0.309
	*P*		0.315	0.450	0.669	0.746	0.692	0.697	0.578
	Han	988							
	Carrier	47	4.87 ± 0.90	1.25 (0.98)	1.79 ± 0.46	2.85 ± 0.45	1.35 ± 0.20	1.03 ± 0.24	1.39 ± 0.37
	Non-carrier	941	4.88 ± 0.86	1.34 (1.09)	1.76 ± 0.54	2.85 ± 0.43	1.31 ± 0.20	1.04 ± 0.24	1.33 ± 0.38
	*F*		0.052	−2.134	0.086	0.026	3.701	0.093	1.562
	*P*		0.820	0.033	0.770	0.872	0.055	0.760	0.212
G-C-G-C-T-A-C-C-A	Jing plus Han	1869							
	Carrier	59	4.99 ± 0.91	1.48 (1.19)	1.77 ± 0.40	2.83 ± 0.44	1.28 ± 0.20	1.05 ± 0.25	1.29 ± 0.38
	Non-carrier	1810	4.97 ± 0.77	1.35 (1.07)	1.77 ± 0.51	2.83 ± 0.38	1.31 ± 0.22	1.04 ± 0.22	1.32 ± 0.38
	*F*		0.775	−3.088	0.090	0.005	5.947	0.296	1.381
	*P*		0.379	0.002	0.765	0.944	0.015	0.587	0.240
	Jing	881							
	Carrier	4	5.14 ± 0.93	1.52 (1.21)	1.71 ± 0.38	2.82 ± 0.44	1.26 ± 0.21	1.06 ± 0.25	1.27 ± 0.38
	Non-carrier	877	5.03 ± 0.76	1.38 (1.09)	1.80 ± 0.46	2.79 ± 0.35	1.31 ± 0.23	1.04 ± 0.22	1.30 ± 0.39
	*F*		1.423	−2.312	4.245	0.648	7.008	1.558	0.566
	*P*		0.233	0.021	0.040	0.421	0.008	0.212	0.452
	Han	988							
	Carrier	55	4.89 ± 0.79	1.34 (1.16)	1.75 ± 0.54	2.89 ± 0.41	1.32 ± 0.19	1.04 ± 0.23	1.33 ± 0.38
	Non-carrier	933	4.87 ± 0.88	1.31 (1.06)	1.86 ± 0.41	2.84 ± 0.44	1.32 ± 0.20	1.04 ± 0.25	1.34 ± 0.38
	*F*		0.021	−1.490	1.910	0.876	0.231	0.198	0.616
	*P*		0.885	0.136	0.167	0.350	0.631	0.657	0.433
G-C-G-C-T-G-C-C-A	Jing plus Han	1869							
	Carrier	199	4.90 ± 0.86	1.30 (1.08)	1.79 ± 0.49	2.80 ± 0.40	1.31 ± 0.23	1.02 ± 0.22	1.34 ± 0.37
	Non-carrier	1670	5.03 ± 0.91	1.42 (1.10)	1.77 ± 0.50	2.85 ± 0.45	1.31 ± 0.21	1.06 ± 0.25	1.31 ± 0.39
	*F*		8.329	−4.113	0.530	4.982	0.028	9.864	2.914
	*P*		0.004	0.000	0.467	0.026	0.867	0.002	0.088
	Jing	881							
	Carrier	133	5.00 ± 0.88	1.35 (1.12)	1.79 ± 0.47	2.78 ± 0.40	1.30 ± 0.22	1.03 ± 0.23	1.31 ± 0.39
	Non-carrier	748	5.18 ± 0.91	1.45 (1.13)	1.77 ± 0.41	2.84 ± 0.43	1.29 ± 0.25	1.08 ± 0.25	1.28 ± 0.38
	*F*		7.709	−2.520	0.013	3.046	0.128	6.670	2.105
	*P*		0.006	0.012	0.909	0.081	0.720	0.010	0.147
	Han	988							
	Carrier	66	4.82 ± 0.83	1.26 (1.05)	1.80 ± 0.55	2.82 ± 0.40	1.32 ± 0.21	1.01 ± 0.22	1.36 ± 0.36
	Non-carrier	922	4.90 ± 0.89	1.37 (1.09)	1.75 ± 0.52	2.87 ± 0.46	1.32 ± 0.20	1.05 ± 0.25	1.33 ± 0.39
	*F*		2.273	−3.327	0.717	2.079	0.015	3.558	0.842
	*P*		0.132	0.001	0.397	0.150	0.904	0.060	0.359
G-C-G-C-T-G-T-C-A	Jing plus Han	1869							
	Carrier	122	4.94 ± 0.86	1.36 (1.16)	1.81 ± 0.53	2.81 ± 0.40	1.31 ± 0.23	1.03 ± 0.23	1.35 ± 0.42
	Non-carrier	1747	5.00 ± 0.90	1.37 (1.07)	1.76 ± 0.48	2.84 ± 0.44	1.31 ± 0.21	1.05 ± 0.25	1.31 ± 0.37
	*F*		1.882	−0.431	1.209	1.204	0.015	3.363	3.350
	*P*		0.170	0.666	0.272	0.273	0.903	0.067	0.067
	Jing	881							
	Carrier	80	5.06 ± 0.86	1.42 (1.11)	1.78 ± 0.46	2.79 ± 0.41	1.30 ± 0.26	1.04 ± 0.24	1.32 ± 0.44
	Non-carrier	801	5.13 ± 0.91	1.44 (1.16)	1.78 ± 0.43	2.82 ± 0.43	1.30 ± 0.22	1.06 ± 0.25	1.28 ± 0.37
	*F*		0.922	−0.637	0.179	0.859	0.144	0.798	0.554
	*P*		0.337	0.524	0.673	0.354	0.704	0.372	0.457
	Han	988							
	Carrier	42	4.83 ± 0.84	1.31 (1.04)	1.83 ± 0.60	2.83 ± 0.40	1.33 ± 0.20	1.01 ± 0.23	1.38 ± 0.41
	Non-carrier	946	4.89 ± 0.88	1.33 (1.17)	1.75 ± 0.51	2.85 ± 0.45	1.32 ± 0.20	1.04 ± 0.25	1.33 ± 0.37
	*F*		1.143	−1.183	2.233	0.372	0.496	2.237	2.717
	*P*		0.285	0.237	0.135	0.542	0.482	0.135	0.100

HDL, high density lipoprotein; LDL, low density lipoprotein.

**Table 7:** Association of the alleles and genotypes of the *DOCK7*, *PCSK9* and *GAALNT2* SNPs and serum lipid traits in the Jing and Han populations.

Lipid	SNP	Affected allele/ Other allele	Affected genotype/ Other genotype	Beta	Std.error	*t*	*P*-value
**Jing plus Han**							
TC	*DOCK7*rs10889332		CC,CT/TT	0.081	0.036	3.161	0.002
	*DOCK7*rs10889332	C/T		0.057	0.047	2.231	0.026
	*PCSK9*rs615563		GG,AG/AA	0.068	0.039	2.634	0.009
	*PCSK9*rs615563	G/A		0.059	0.048	2.292	0.022
	*PCSK9* rs7552841		CC,CT/TT	0.108	0.041	4.234	0.000
	*PCSK9* rs7552841	C/T		0.101	0.049	3.941	0.000
	*GALNT2*rs2760537		CC,CT/TT	0.055	0.034	2.154	0.031
	*GALNT2*rs11122316			−0.055	0.047	−2.145	0.032
TG	*DOCK7* rs1168013		GG,CG/CC	0.087	0.033	3.426	0.001
	*DOCK7*rs10889332		CC,CT/TT	0.099	0.034	3.903	0.000
	*PCSK9*rs615563		GG,AG/AA	0.094	0.035	3.995	0.000
	*PCSK9*rs7552841		CC,CT/TT	0.126	0.037	5.325	0.000
	*PCSK9*rs11206517	T/G		−0.056	0.035	−2.155	0.031
	*GALNT2*rs1997947		GG,AG/AA	0.083	0.035	3.440	0.001
	*GALNT2*rs2760537		CC,CT/TT	0.114	0.030	4.811	0.000
	*GALNT2*rs4846913		CC,AC/AA	0.104	0.036	4.384	0.000
	*GALNT2*rs4846913	C/A		−0.109	0.027	−4.235	0.000
	*GALNT2*rs11122316		GG,AG/AA	0.119	0.031	4.978	0.000
HDL cholesterol	*PCSK9*rs615563	G/A		0.052	0.024	1.988	0.047
	*PCSK9* rs7552841	C/T		0.100	0.025	3.816	0.000
	*PCSK9*rs11206517		TT,GT/GG	−0.067	0.032	−2.573	0.010
	*GALNT2*rs1997947		GG,AG/AA	−0.081	0.021	−3.111	0.002
	*GALNT2*rs4846913		GG,AG/AA	−0.078	0.022	−3.014	0.003
LDL cholesterol	*DOCK7*rs10889332		CC,CT/TT	0.069	0.018	2.617	0.009
	*PCSK9*rs615563		GG,AG/AA	0.068	0.020	2.595	0.010
	*PCSK9*rs615563	G/A		0.052	0.024	1.988	0.047
	*PCSK9* rs7552841		CC,CT/TT	0.114	0.021	4.337	0.000
	*PCSK9*rs7552841	C/T		0.100	0.025	3.816	0.000
ApoA1	*DOCK7* rs1168013	G/C		0.053	0.011	2.050	0.041
	*DOCK7*rs10889332		CC,CT/TT	0.065	0.009	2.485	0.013
	*GALNT2*rs1997947		GG,AG/AA	−0.128	0.010	−4.905	0.000
	*GALNT2*rs1997947	G/A		−0.089	0.012	−3.434	0.001
	*GALNT2*rs4846913		CC,AC/AA	−0.117	0.010	−4.488	0.000
	*GALNT2*rs4846913	G/A		−0.128	0.012	−4.901	0.000
ApoB	*DOCK7*rs10889332		CC,CT/TT	0.103	0.010	4.023	0.000
	*DOCK7*rs10889332	C/T		0.060	0.013	2.341	0.019
	*PCSK9*rs615563		GG,AG/AA	0.091	0.011	3.572	0.000
	*PCSK9*rs615563	G/A		0.093	0.013	3.598	0.000
	*PCSK9*rs7552841		CC,CT/TT	0.175	0.011	6.843	0.000
	*PCSK9*rs7552841	C/T		0.164	0.014	6.386	0.000
ApoA1/ApoB	*PCSK9*rs615563		GG,AG/AA	−0.073	0.016	−2.860	0.004
	*PCSK9*rs615563	G/A		−0.087	0.020	−3.420	0.001
	*PCSK9*rs7552841		CC,CT/TT	−0.115	0.017	−4.543	0.000
	*PCSK9*rs7552841	C/T		−0.112	0.020	−4.390	0.000
	*GALNT2*rs1997947		GG,AG/AA	−0.089	0.016	−3.443	0.001
	*GALNT2*rs1997947	G/A		−0.060	0.020	−2.321	0.020
	*GALNT2*rs4846913		CC,AC/AA	−0.088	0.017	−3.483	0.001
	*GALNT2*rs4846913	C/A		−0.095	0.020	−3.747	0.000
**Jing**							
TC	*DOCK7* rs1168013		GG,CG/CC	−0.109	0.054	−2.819	0.005
	*DOCK7*rs10889332		CC,CT/TT	0.111	0.054	2.823	0.005
	*PCSK9*rs7552841		CC,CT/TT	0.116	0.058	3.159	0.002
	*PCSK9*rs7552841	C/T		0.132	0.072	3.561	0.000
	*GALNT2*rs2760537		CC,CT/TT	0.079	0.049	2.138	0.033
	*GALNT2*rs11122316	G/A		−0.076	0.071	−2.048	0.041
TG	*DOCK7* rs1168013		GG,CG/CC	0.089	0.045	2.512	0.012
	*DOCK7* rs1168013	G/C		0.115	0.060	3.335	0.001
	*DOCK7*rs10889332		CC,CT/TT	0.089	0.046	2.460	0.014
	*PCSK9*rs615563		GG,AG/AA	0.072	0.048	2.131	0.034
	*PCSK9*rs7552841		CC,CT/TT	0.141	0.049	4.169	0.000
	*PCSK9*rs7552841	C/T		0.139	0.062	4.022	0.000
	*GALNT2*rs1997947		GG,AG/AA	0.104	0.048	3.015	0.003
	*GALNT2*rs1997947	G/A		0.107	0.061	3.048	0.002
	*GALNT2*rs2760537		CC,CT/TT	0.140	0.042	4.111	0.000
	*GALNT2*rs2760537	C/T		0.116	0.061	3.337	0.001
	*GALNT2*rs4846913		CC,AC/AA	0.095	0.049	2.806	0.005
	*GALNT2*rs4846913	C/A		0.103	0.061	3.004	0.003
	*GALNT2*rs11122316		GG,AG/AA	0.124	0.043	3.605	0.000
	*GALNT2*rs11122316	G/A		0.117	0.062	3.342	0.001
HDL cholesterol	*GALNT2*rs1997947		GG,AG/AA	−0.102	0.027	−2.766	0.006
	*GALNT2*rs11122316	G/A		−0.106	0.034	−2.916	0.004
LDL cholesterol	*DOCK7* rs1168013		GG,CG/CC	−0.139	0.026	−3.487	0.001
	*DOCK7*rs10889332		CC,CT/TT	0.129	0.026	3.217	0.001
	*PCSK9*rs7552841		CC,CT/TT	0.145	0.028	3.836	0.000
	*PCSK9*rs7552841	C/T		0.155	0.035	4.052	0.000
ApoA1	*PCSK9*rs11206517	T/G		0.076	0.023	1.987	0.047
	*GALNT2*rs1997947		GG,AG/AA	−0.157	0.014	−4.121	0.000
	*GALNT2*rs1997947	G/A		−0.129	0.018	−3.390	0.001
	*GALNT2*rs4846913		CC,AC/AA	−0.076	0.015	−2.010	0.045
	*GALNT2*rs4846913	C/A		−0.090	0.018	−2.369	0.018
ApoB	*DOCK7* rs1168013		GG,CG/CC	−0.112	0.014	−2.923	0.004
	*DOCK7* rs1168013	G/C		−0.110	0.019	−2.857	0.004
	*DOCK7*rs10889332		CC,CT/TT	0.175	0.014	4.502	0.000
	*DOCK7*rs10889332	C/T		0.125	0.019	3.200	0.001
	*PCSK9*rs7552841		CC,CT/TT	0.221	0.015	6.079	0.000
	*PCSK9*rs7552841	C/T		0.236	0.019	6.435	0.000
	*PCSK9*rs11206517		TT,GT/GG	0.093	0.022	2.444	0.015
	*PCSK9*rs11206517	T/G		0.085	0.025	2.222	0.027
ApoA1/ApoB	*PCSK9*rs615563	G/A		−0.075	0.029	−2.017	0.044
	*DOCK7*rs10889332		CC,CT/TT	−0.080	0.021	−2.154	0.032
	*PCSK9*rs7552841		CC,CT/TT	−0.177	0.024	−4.770	0.000
	*PCSK9*rs7552841	C/T		−0.183	0.030	−4.905	0.000
	*GALNT2*rs1997947		GG,AG/AA	−0.098	0.023	−2.597	0.000
	*GALNT2*rs1997947	G/A		−0.074	0.029	−1.968	0.050
**Han**							
TC	*DOCK7* rs1168013		GG,CG/CC	0.101	0.047	2.831	0.005
	*DOCK7* rs1168013	G/C		0.103	0.061	2.902	0.004
	*PCSK9*rs7552841		CC,CT/TT	0.076	0.058	2.143	0.032
TG	*DOCK7* rs1168013		GG,CG/CC	0.084	0.049	2.316	0.021
	*DOCK7*rs10889332		CC,CT/TT	0.111	0.052	3.083	0.002
	*DOCK7*rs10889332	C/T		0.116	0.060	3.435	0.001
	*PCSK9*rs615563		GG,AG/AA	0.123	0.052	3.661	0.000
	*PCSK9*rs615563	G/A		0.153	0.064	4.476	0.000
	*PCSK9*rs7552841		CC,CT/TT	0.119	0.056	3.509	0.000
	*PCSK9*rs7552841	C/T		0.100	0.065	2.933	0.003
	*PCSK9*rs11206517		TT,GT/GG	0.105	0.079	3.121	0.002
	*PCSK9*rs11206517	T/G		0.076	0.088	2.248	0.025
	*GALNT2*rs2760537		CC,CT/TT	0.090	0.043	2.717	0.007
	*GALNT2*rs2760537	C/T		0.078	0.059	2.312	0.021
	*GALNT2*rs4846913		CC,AC/AA	0.120	0.052	3.640	0.000
	*GALNT2*rs4846913	C/A		0.122	0.064	3.618	0.000
	*GALNT2*rs11122316		GG,AG/AA	0.121	0.044	3.666	0.000
	*GALNT2*rs11122316	G/A		0.090	0.060	2.690	0.007
HDL cholesterol	*DOCK7* rs1168013	G/C		0.084	0.037	2.351	0.019
	*PCSK9*rs11206517		TT,GT/GG	−0.112	0.052	−3.044	0.002
	*PCSK9*rs11206517	T/G		−0.132	0.056	−3.673	0.000
	*GALNT2*rs4846913		CC,AC/AA	−0.116	0.033	−3.262	0.001
	*GALNT2*rs4846913	C/A		−0.146	0.040	−4.111	0.000
LDL cholesterol	*DOCK7* rs1168013		GG,CG/CC	0.107	0.024	2.965	0.003
	*DOCK7* rs1168013	G/C		0.093	0.032	2.567	0.010
ApoA1	*DOCK7* rs1168013	G/C		0.109	0.014	3.094	0.002
	*DOCK7*rs10889332		CC,CT/TT	0.082	0.012	2.315	0.021
	*PCSK9*rs11206517		TT,GT/GG	−0.114	0.020	−3.138	0.002
	*PCSK9*rs11206517	T/G		−0.125	0.022	−3.448	0.001
	*GALNT2*rs1997947		GG,AG/AA	−0.079	0.013	−2.231	0.026
	*GALNT2*rs4846913		CC,AC/AA	−0.164	0.013	−4.627	0.000
	*GALNT2*rs4846913	C/A		−0.176	0.016	−5.000	0.000
ApoB	*DOCK7* rs1168013		GG,CG/CC	0.119	0.013	3.314	0.001
	*DOCK7* rs1168013	G/C		0.101	0.018	2.797	0.005
	*PCSK9*rs615563		GG,AG/AA	0.087	0.015	2.406	0.016
	*PCSK9*rs615563	G/A		0.080	0.019	2.200	0.028
	*PCSK9*rs7552841		CC,CT/TT	0.104	0.017	2.868	0.004
ApoA1/ApoB	*DOCK7* rs1168013		GG,CG/CC	−0.073	0.020	−2.074	0.038
	*PCSK9*rs615563	G/A		−0.084	0.028	−2.394	0.017
	*PCSK9*rs11206517		TT,GT/GG	−0.072	0.036	−2.027	0.043
	*GALNT2*rs1997947		GG,AG/AA	−0.110	0.023	−3.139	0.002
	*GALNT2*rs4846913		CC,AC/AA	−0.118	0.023	−3.363	0.001
	*GALNT2*rs4846913	C/A		−0.136	0.028	−3.908	0.000

HDL, high density lipoprotein; LDL, low density lipoprotein; Association of serum lipid traits and allele and genotypes in Jing, Han and combined the Jing and Han populations were assessed by multivariable linear regression analyses with stepwise modeling.
