# Supplementary material for: Association between the DOCK7, PCSK9 and GALNT2 Gene Polymorphisms and Serum Lipid levels
Source: Sci Rep. 2016 Jan 8;6:19079. doi: 10.1038/srep19079 (PMC4705473; doi:10.1038/srep19079)
Supplement: Supplemental Tables 1 and 2 [file srep19079-s1.doc]

**Association between the DOCK7, PCSK9 and GALNT2 Gene Polymorphisms and Serum Lipid levels**

Tao Guo ^1^, Rui-Xing Yin ^1^, Feng Huang ^1^, Li-Mei Yao ^1^, Wei-Xiong Lin ^2^ & Shang-Ling Pan ^3^

**Supplemental Table 1.** Characteristics of the *DOCK7, PCSK9 and GALNT2* SNPs.

| **SNP ID (rs#)** | **HUGO name** | **Chromosome** | **Position** | **Gene region** | **MAF/Minor allele count** |
| --- | --- | --- | --- | --- | --- |
| ***DOCK7*** |  |  |  |  |  |
| rs1168013 | NM_001271999.1:c.3612-1721G>C | 1 | 62769426 | Intron region | C=0.4319/2163 |
| rs10889332 | NM_001271999.1:c.5466+3747G>A | 1 | 62723446 | intron region | T=0.3984/1995 |
| ***PCSK9*** |  |  |  |  |  |
| rs615563 | NM_174936.3:c.1682-752A>G | 1 | 55298884 | intron region | A=0.3195/1600 |
| rs7552841 | NM_174936.3:c.799+288C>T | 1 | 55291340 | intron region | T=0.2750/1377 |
| rs11206517 | NM_174936.3:c.1682-620T>G | 1 | 55299016 | intron region | G=0.0803/402 |
| ***GALNT2*** |  |  |  |  |  |
| rs1997947 | NM_001291866.1:c.13-30200G>A | 1 | 228350387 | intron region | G=0.3043/1524 |
| rs2760537 | NM_001291866.1:c.106+12355G>A | 1 | 228393035 | intron region | A=0.2574/1289 |
| rs4846913 | NM_001291866.1:c.13-19249C>A | 1 | 228361338 | intron region | A=0.3265/1635 |
| rs11122316 | NM_001291866.1:c.12+43278A>G | 1 | 228303726 | intron region | G=0.4716/2362 |

**Supplemental Table 2.** The sequences of forward and backward primers, and restriction enzymes for genotyping of the *DOCK7, PCSK9 and GALNT2* SNPs.

| **SNP** | **Primer sequence** | **Annealing temperature** | **PCR product** | **Restriction enzyme/site** | **Restriction fragments (bp)** | **Allele** |
| --- | --- | --- | --- | --- | --- | --- |
| ***DOCK7*** |  |  |  |  |  |  |
| rs1168013 | AGGGTCGGTGGGTGGAAA | 60 °C | 365bp | HaeIII [G] | 365 | C |
|  | ATGGGATTGAGCAATGTAAGCA |  |  |  | 186+169 | G |
| rs10889332 | GCAGGTGGATTGCTTGGG | 60 °C | 368bp | TaqI [C] | 368 | T |
|  | TTTCGGGAGTCATGTGGC |  |  |  | 279+89 | C |
| ***PCSK9*** |  |  |  |  |  |  |
| rs615563 | TGCTCTAATCACGCTCCC | 56 °C | 365bp | HaeIII [G] | 365 | A |
|  | CCATTGGCTAAGAAACCTAAAA |  |  |  | 233+32 | G |
| rs7552841 | AGGGAAGGGCACGGTTAG | 60 °C | 496bp | MspI [C] | 496 | T |
|  | TGCCAGTTCCTCCACCAC |  |  |  | 320+176 | C |
| rs11206517 | TGCTCTAATCACGCTCCC | 56 °C | 367bp | MvaI [G] | 367 | T |
|  | CCATTGGCTAAGAAACCTAAAA |  |  |  | 201+166 | G |
| ***GALNT2*** |  |  |  |  |  |  |
| rs1997947 | TTGCTTGTTGGAGGTTGG | 62 °C | 480bp | TaqI [G] | 480 | A |
|  | AGGAAGGGACTGTGCTGA |  |  |  | 352+128 | G |
| rs2760537 | CTGGCTGGAACCCCTCTTTA | 56 °C | 249+55bp | HaeIII [C] | 249+55 | T |
|  | ACACGCCCATCTCTCTTTCA |  |  |  | 217+32+55 | C |
| rs4846913 | CGCCACCTCCCATCACAGA | 60 °C | 436bp | PstI [C] | 436 | A |
|  | AAGCCTCACATCAACAGCAAAG |  |  |  | 243+193 | C |
| rs11122316 | CACAGTGGTCCCGTAAGA | 60°C | 456bp | TaqI [G] | 456 | A |
|  | GGCATAAGCTCCAGAGGC |  |  |  | 428+28 | G |
